# Supplementary material for: Synergism between CMG helicase and leading strand DNA polymerase at replication fork
Source: Nat Commun. 2023 Sep 20;14:5849. doi: 10.1038/s41467-023-41506-0 (PMC10511561; doi:10.1038/s41467-023-41506-0)
Supplement: Supplementary file 1 — Supplementary Information [file 41467_2023_41506_MOESM1_ESM.pdf]

Supplementary Information

Synergism between CMG Helicase and Leading Strand DNA Polymerase at Replication Fork

Zhichun Xu[1*], Jianrong Feng[2*], Daqi Yu[2*], Yunjing, Huo[1*], Xiaohui Ma[2*], Wai Hei Lam[1], Zheng Liu[3], Xiang David Li[3], Toyotaka Ishibashi[2], Shangyu Dang[2,4,5#] & Yuanliang Zhai[1#]

[*]These authors contributed equally to this work

[#]Co-corresponding authors

This document contains 10 Figures and 2 Tables

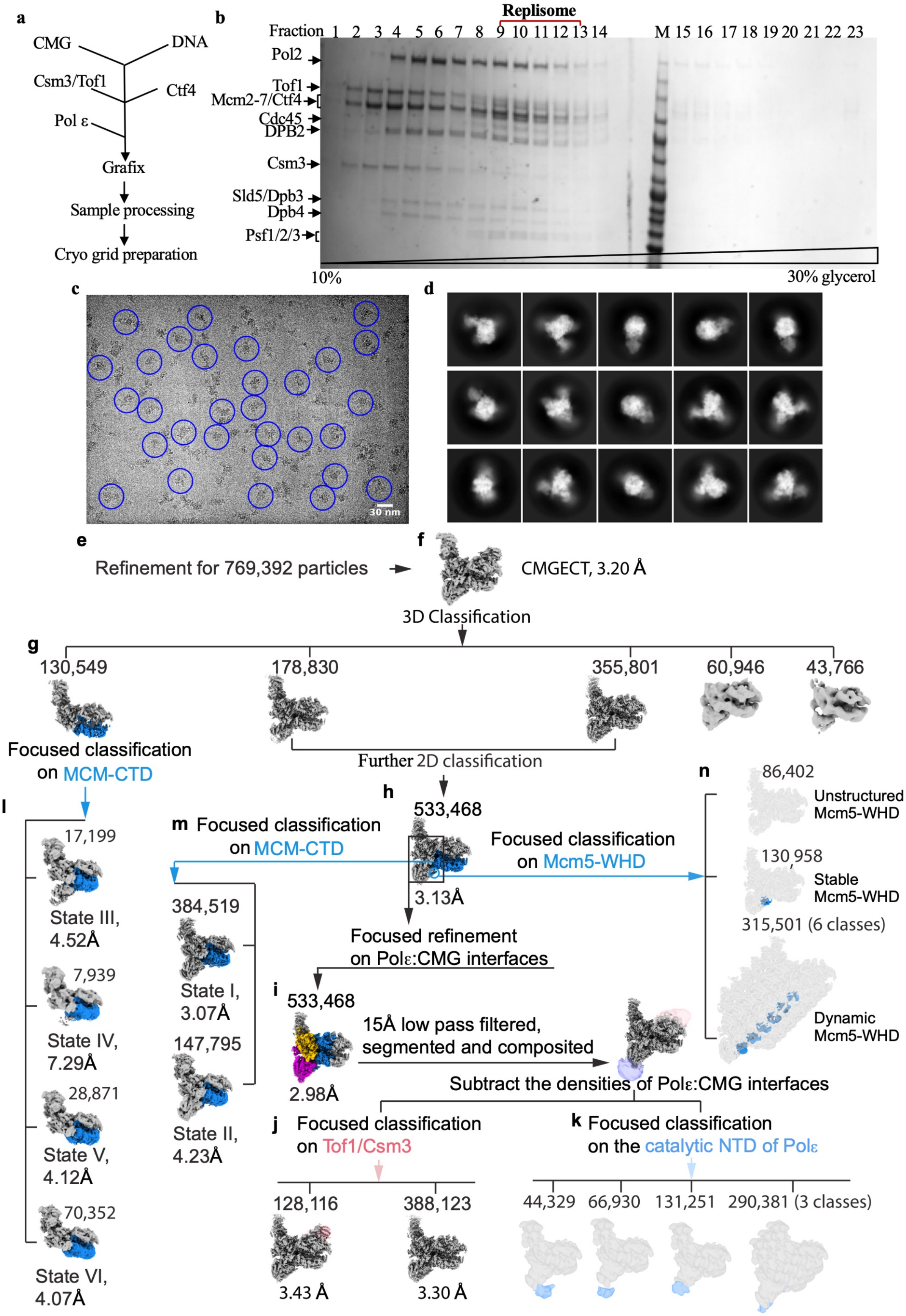
a
CMG
DNA
Csm3/Tof1
Ctf4
Pol ε
Grafix
Sample processing
Cryo grid preparation
b
Replisome
Fraction 1 2 3 4 5 6 7 8 9 10 11 12 13 14 M 15 16 17 18 19 20 21 22 23
Pol2
Tof1
Mcm2-7/Ctf4
Cdc45
DPB2
Csm3
Sld5/Dpb3
Dpb4
Psf1/2/3
10%
30% glycerol
c
30 nm
d
e
Refinement for 769,392 particles
f
CMGECT, 3.20 Å
3D Classification
g
130,549
178,830
355,801
60,946
43,766
Focused classification on MCM-CTD
Further 2D classification
n
86,402
Unstructured Mcm5-WHD
130,958
Stable Mcm5-WHD
315,501 (6 classes)
Dynamic Mcm5-WHD
l
17,199
State III, 4.52Å
7,939
State IV, 7.29Å
28,871
State V, 4.12Å
70,352
State VI, 4.07Å
h
533,468
3.13Å
m Focused classification on MCM-CTD
384,519
State I, 3.07Å
147,795
State II, 4.23Å
Focused classification on Mcm5-WHD
Focused refinement on Polε:CMG interfaces
i
533,468
2.98Å
15Å low pass filtered, segmented and composited
Subtract the densities of Polε:CMG interfaces
j Focused classification on Tof1/Csm3
128,116
3.43 Å
388,123
3.30 Å
k Focused classification on the catalytic NTD of Polε
44,329
66,930
131,251
290,381 (3 classes)

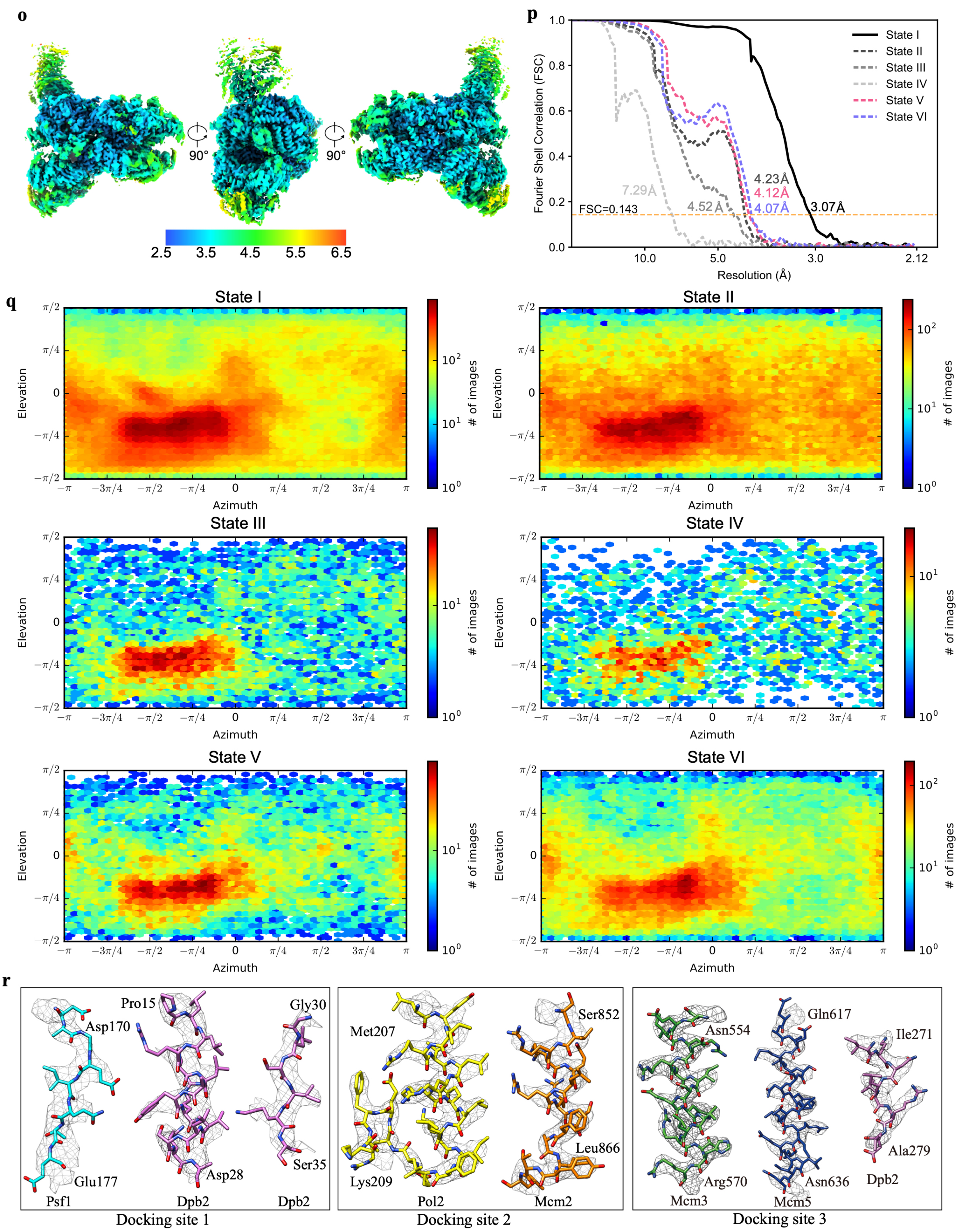

Supplementary Figure 1. Sample preparation and structure determination of the replisome complexes.

(**a**) A flowchart of the procedure for cryo sample preparation.

(**b**) Replisome complexes were subjected to 10-30% glycerol gradient centrifugation. Fractions were collected, resolved on SDS-PAGE and visualized by silver staining. This analysis showed that peak fractions (9-13) contain intact replisome complexes. Based on this result, similar

fractions containing crosslinked samples after grafix were pooled and processed for further EM analysis.

(**c**) A representative raw micrograph of cryo-EM data with replisome particles highlighted in blue circle.

(**d**) Representative 2D class averages of cryo-EM particles from reference-free classification.

(**e-n**) Workflow of image processing of the replisome particles. See Materials and Methods for details.

(**o**) Different views of the cryo-EM density map of the replisome (State I) colour-coded to indicate the range of the local resolution.

(**p**) FSC curves of the final density maps for the indicated replisome complexes in each state.

(**q)** Euler angle orientation distribution of the final particles in each state.

(**r**) Cryo-EM densities for indicated regions of the replisome.

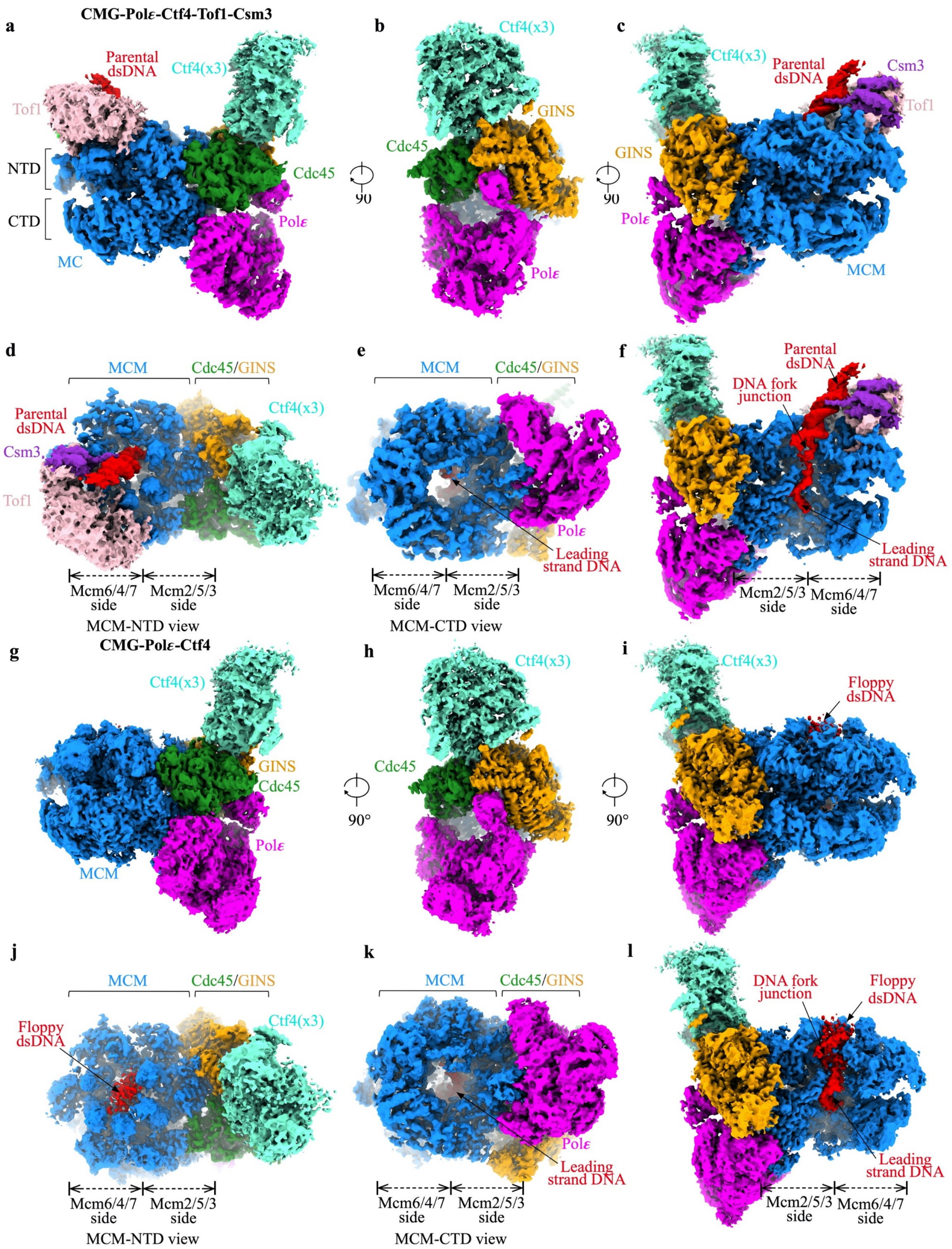

Supplementary Figure 2. Overall structure of a leading-strand replisome at replication fork.

(**a**-**c**) Side views of cryo-EM density map of the replisome CMG-Polε-Ctf4-Tof1-Csm3 at a preformed forked DNA, displayed with indicated rotations.

(**d**, **e**) Top NTD (**d**) and bottom CTD (**e**) of MCM views of the replisome structure.

(**f**) Same as (**c**) but with Mcm3 and Mcm7 removed to highlight the conformation of fork DNA inside the MCM ring.
(**g-l**) Same as (**a-i**) but with the absence of Tof1 and Csm3 highlighting a flexible parental duplex DNA.
MCM ring, Cdc45, GINS, Polε, Ctf4, Tof1, Csm3 and DNA are color coded and labelled as indicated.

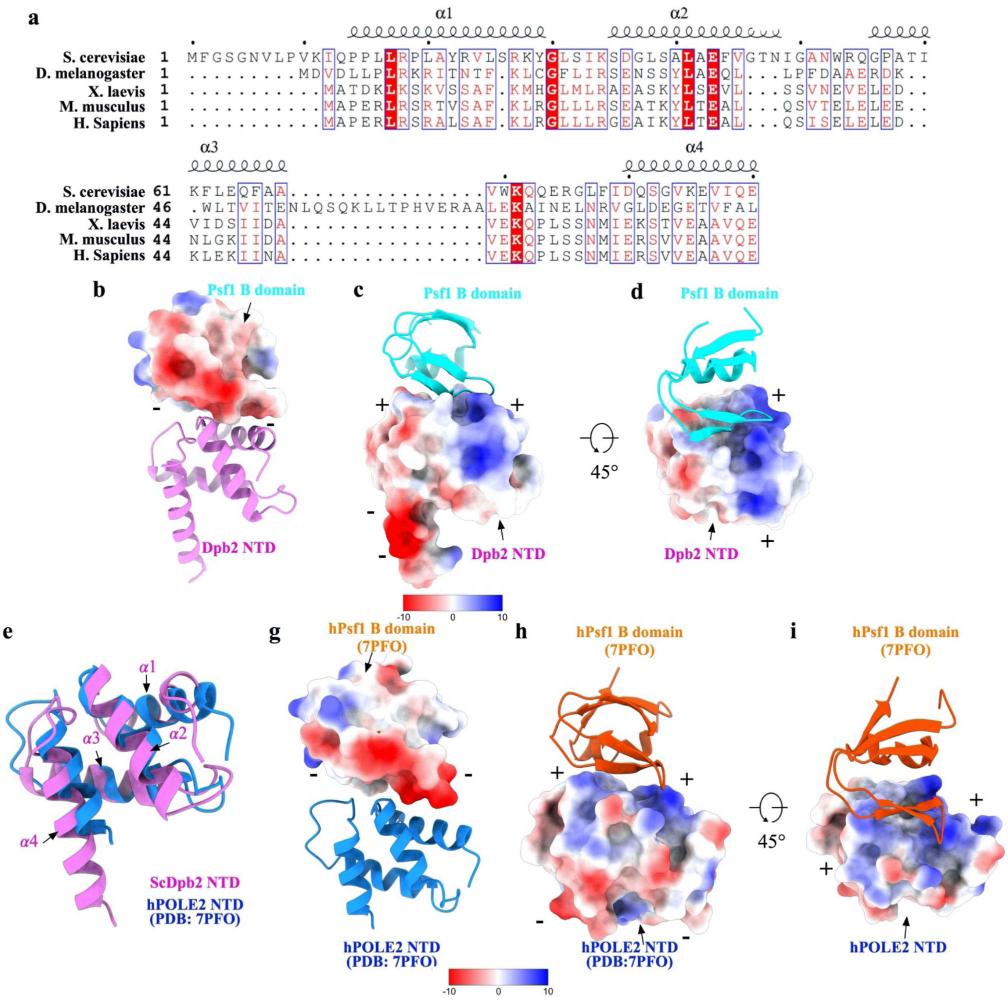

Supplementary Figure 3. Electrostatic analysis of Dpb2-Psf1 interface.

(**a**) Multiple sequence alignment of Dpb2-NTD from various species as indicated. The locations of α helices are labelled at top.

(**b-d**) Interaction between Psf1 B domain and Dpb2-NTD. The electrostatic surface potential maps of these two domains, calculated are displayed in (**b**) and (**c**)respectively. (**c**) is same as (**b**) but with indicated rotation, highlighting a polarized pattern of the surface charge on Dpb2-NTD.

(**e**) hPOLE2-NTD from a human replisome structure (PDB: 7PFO) is superimposed with ScDpb2-NTD from our yeast replisome structure.

(**f-h**) same as (**a-c**) but shown with hPOLE2-NTD and hPsf1 B domain (PDB: 7PFO).

The electrostatic potentials were calculated with the APBS plugin in ChimeraX using the default settings and displayed with a range of ± 10 kT/e.

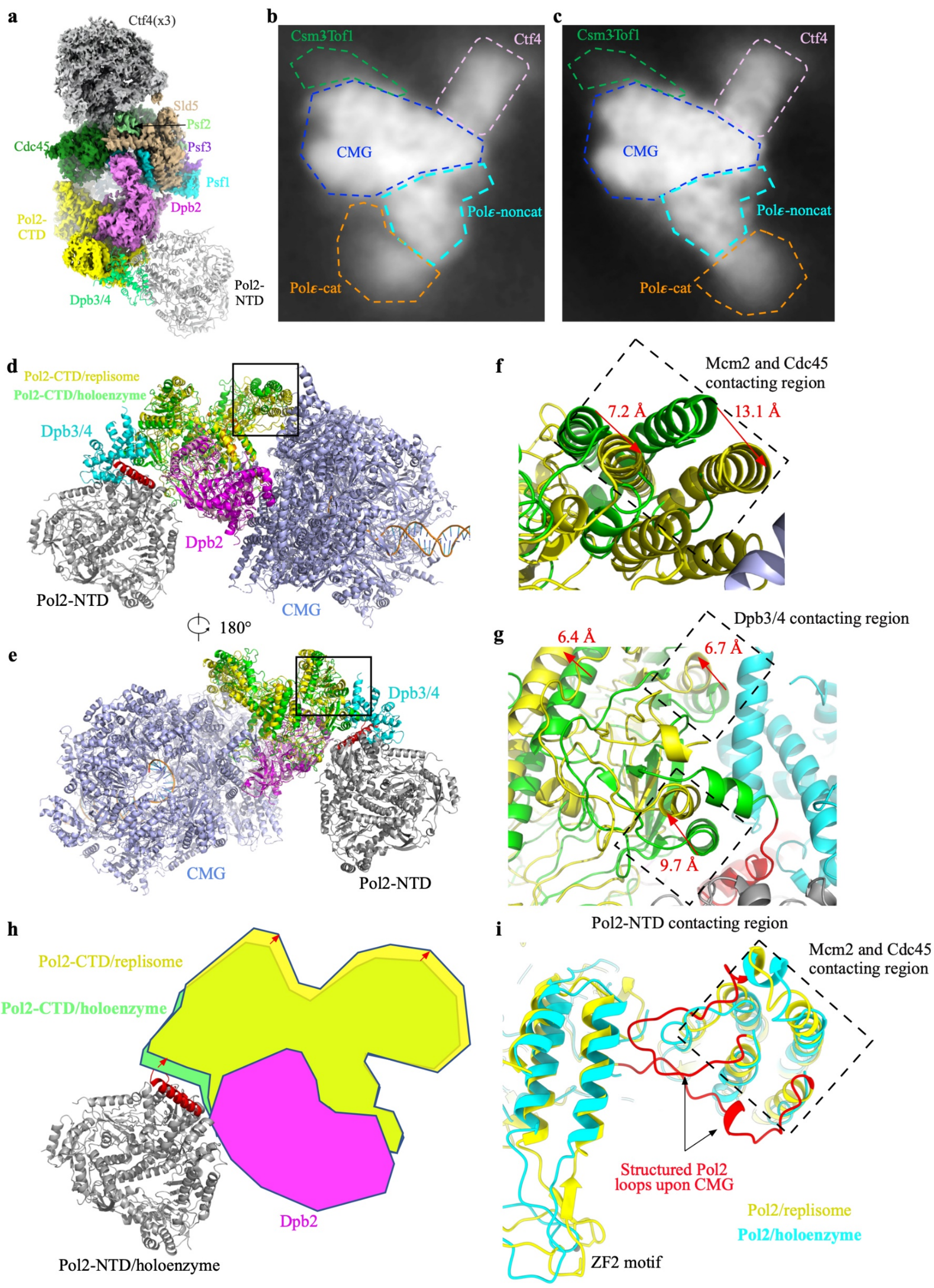

Supplementary Figure 4. Conformational changes in Pol2 upon MCM binding.

(**a**) Density map of the leading-strand replisome superimposed with the atomic model of Polε holoenzyme, highlighting a flexible Pol2-NTD and Dpb3/4 in the replisome.

(**b**, **c**) Two representative 2D average images showing diffuse signals of Pol2 catalytic NTD.

(**d**, **e**) Superimposition of Polε structures from the leading-strand replisome and the holoenzyme using Dpb2 as a reference. Relevant subunits and domains are color coded and labelled as indicated.

(**f**, **g**) Zoomed-in views of the boxed regions in (**d**) and (**e**). Conformational changes exemplified by displacement of several helices are marked by red arrows.

(**h**) Schematic illustration of rotational shift in Pol2-CTD induced by Polε binding to MCM ring.

(**i**) Upon MCM binding, Pol2-M2C45BD flanking loops (red) become structured. Mcm2 and Cdc45 contacting region in Pol2 is boxed.

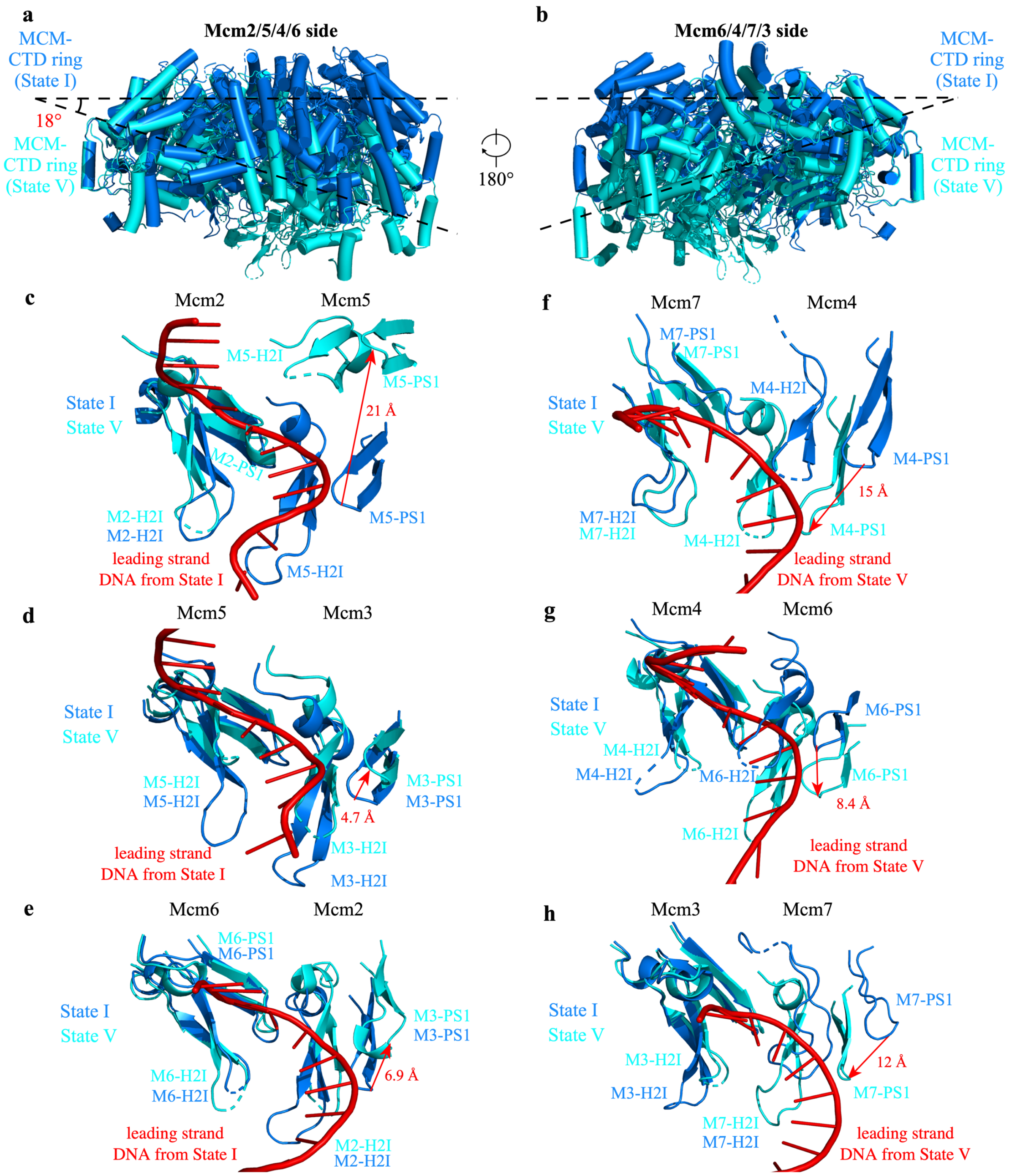

Supplementary Figure 5. DNA translocation driven by displacement of ATPase loops around the motor domains of the MCM ring.

(**a**, **b**) Superimposition of the MCM-CTD rings from State I and State V using Mcm3 as a reference, highlighting the orientation change of the MCM motor domain.

(**c-h**) Comparison of H2I and PS1 loops of each inter-subunit interface from State I (blue) and State V (cyan) showing movement of ATPase loops for DNA threading. Loops of the left subunit used as a reference for superimposition. For clarity, DNA from State V replisome is

not shown in (**c-e**); and DNA from State I not shown in (**f-h**). The directions and distances of PS1 displacements are marked with red arrows.

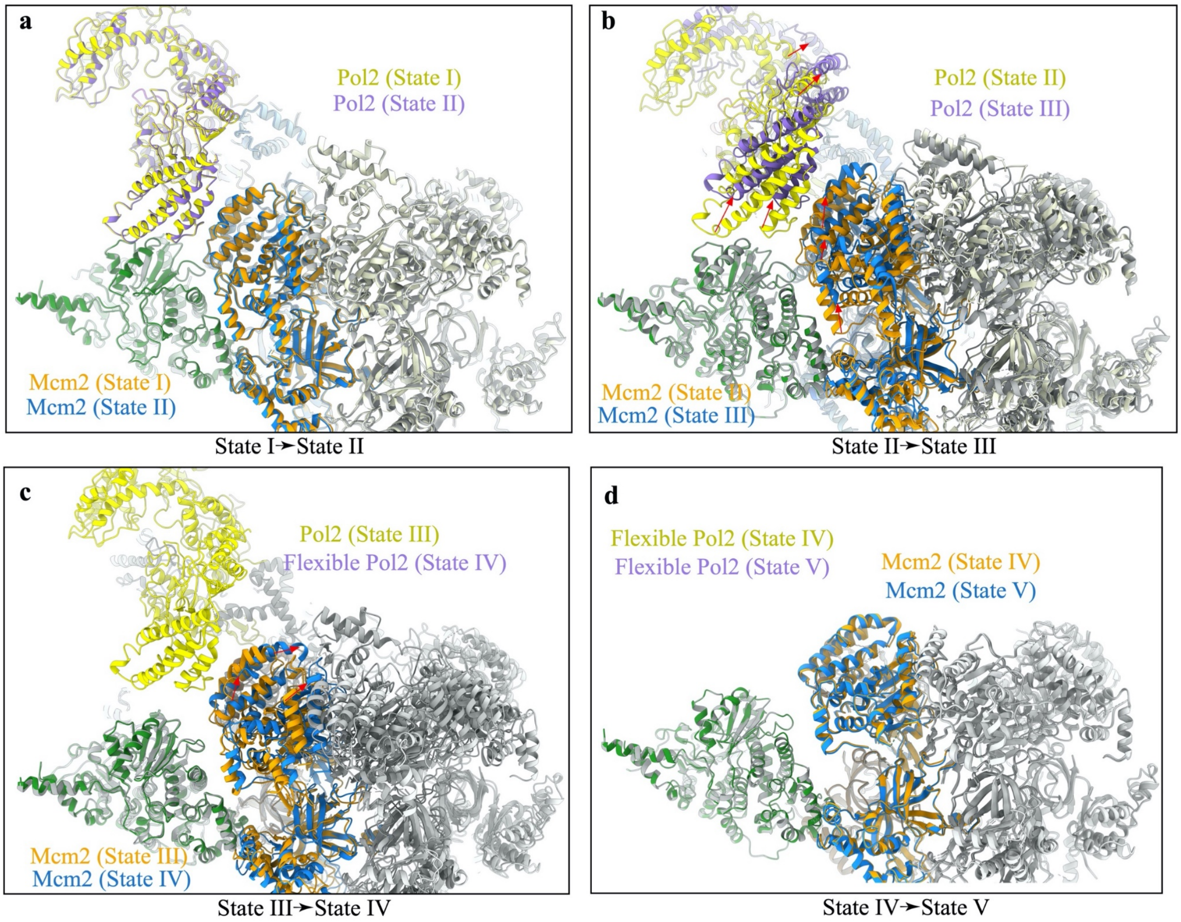

Supplementary Figure 6. Polε stability correlated with rotational movement of DNA around MCM pore.

(**a-f**) Superimposition of indicated structures with Cdc45 as a reference, illustrating conformational changes in Pol2 and Mcm2-CTD between the indicated states of replisome as highlighted by red arrows.

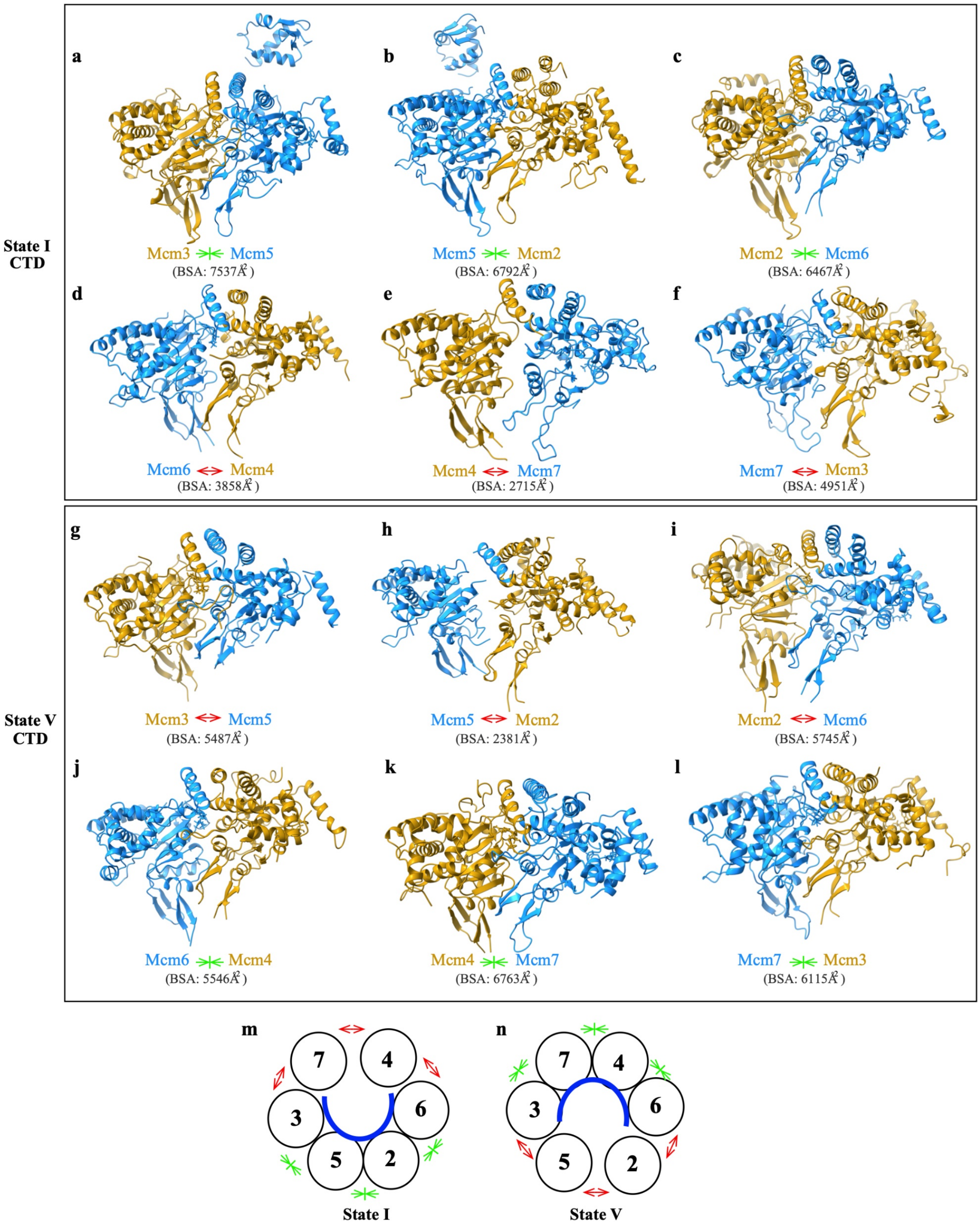

Supplementary Figure 7. DNA translocation and CTD interfaces around MCM ring. Inter-CTD interactions around the MCM rings from the replisomes State I (**a-f**) and State V (**g-l**). The buried surface areas (BSAs) of their interfaces are labeled accordingly. (**m-n**) Schematic organization of CTD interfaces and DNA around the MCM ring for State I (**m**) and State V (**n**).

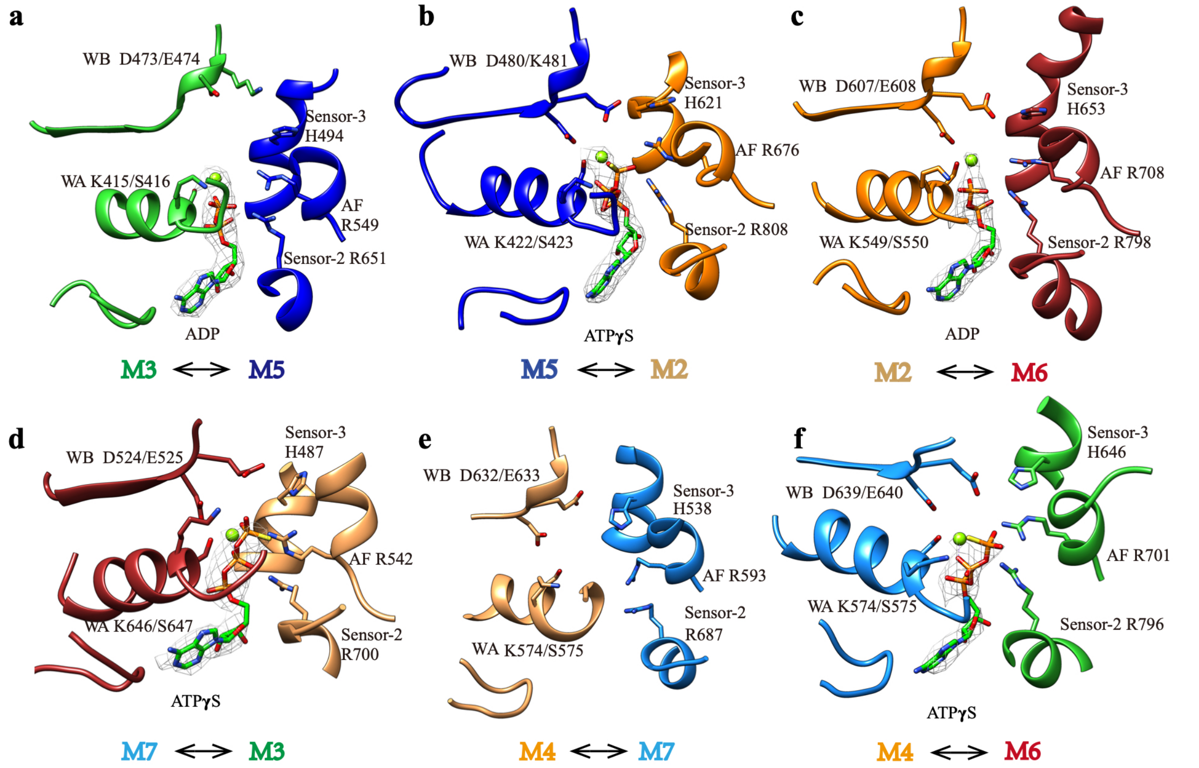

Supplementary Figure 8. Nucleotide occupancy at the ATPase centers of MCM ring in State I replisome.

(**a-f**) The active centers for each MCM dimer from State I replisome with the conserved ATPase elements labelled. Segmented nucleotide densities (gray mesh) were superimposed. Mg^{2+} is shown as green sphere.

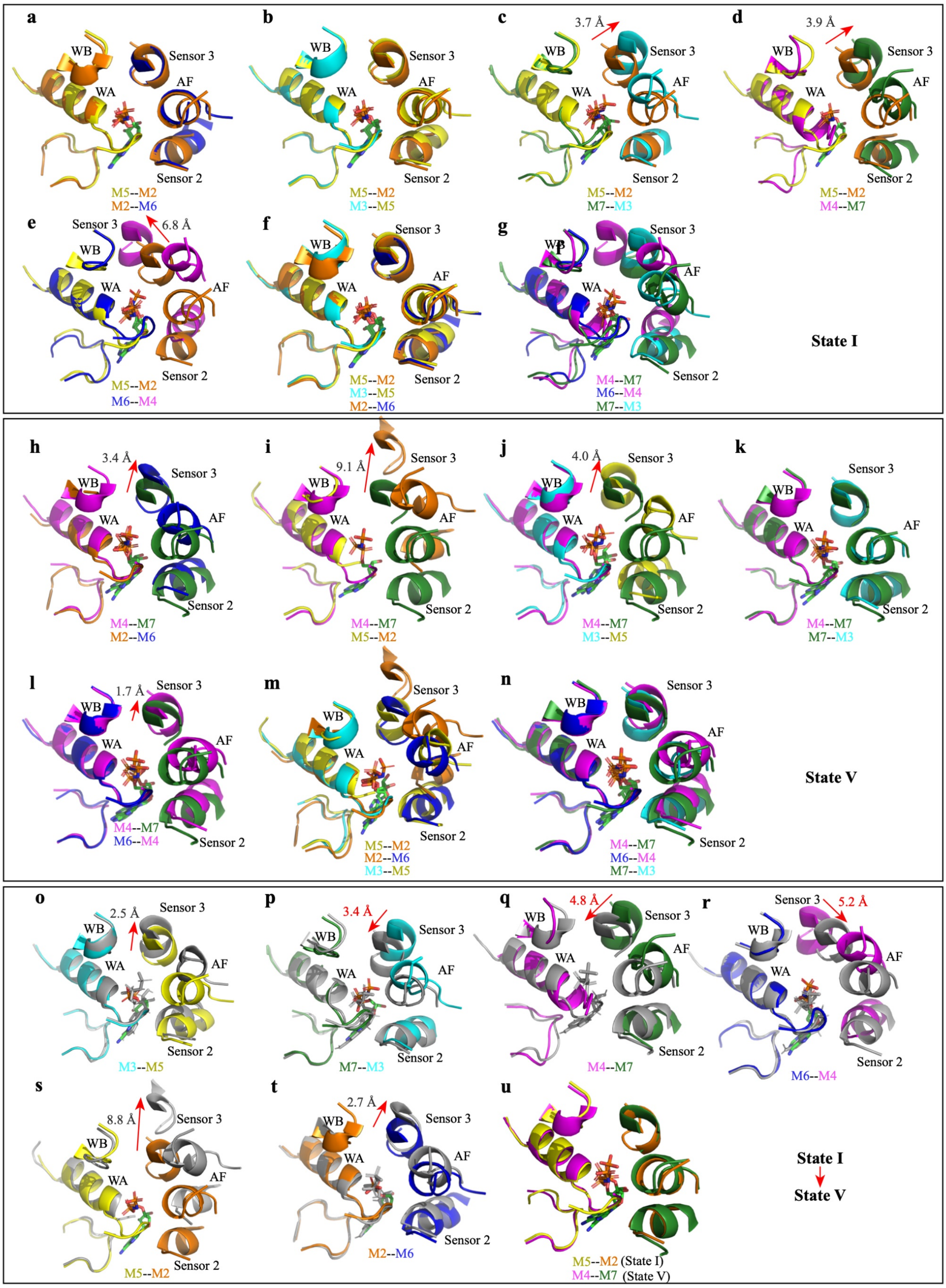

Supplementary Figure 9. ATPase active center configurations at inter-subunit interfaces of the MCM ring.

(**a-e**) Superimposition of ATP-binding sites of Mcm5:2 with other indicated the centers from State I replisome. The walker A and B (WA and WB, respectively) of the left subunit and

sensor 3, sensor 2 and arginine finger (AF) of the right subunit are displayed in cartoon presentation.

(**f-g**) Superimposition of three compact ATPase centers from 3:5, 5:2 and 2:6 (**f**) and three loose ATPase centers from 4:7, 6:4 and 7:3 (**g**).

(**h-n**) same as (**a-g**) but with the centers from State V replisome.

(**o-t**) Comparison of each ATPase center from State I and State V highlighting their conformational differences.

(**u**) Superimposition of the center of 5:2 (State I) with that of 4:7 (State V)

Walker A and B motifs are used as a reference for alignment in all panels. The directions and distances of sensor 3 movements are marked with red arrows.

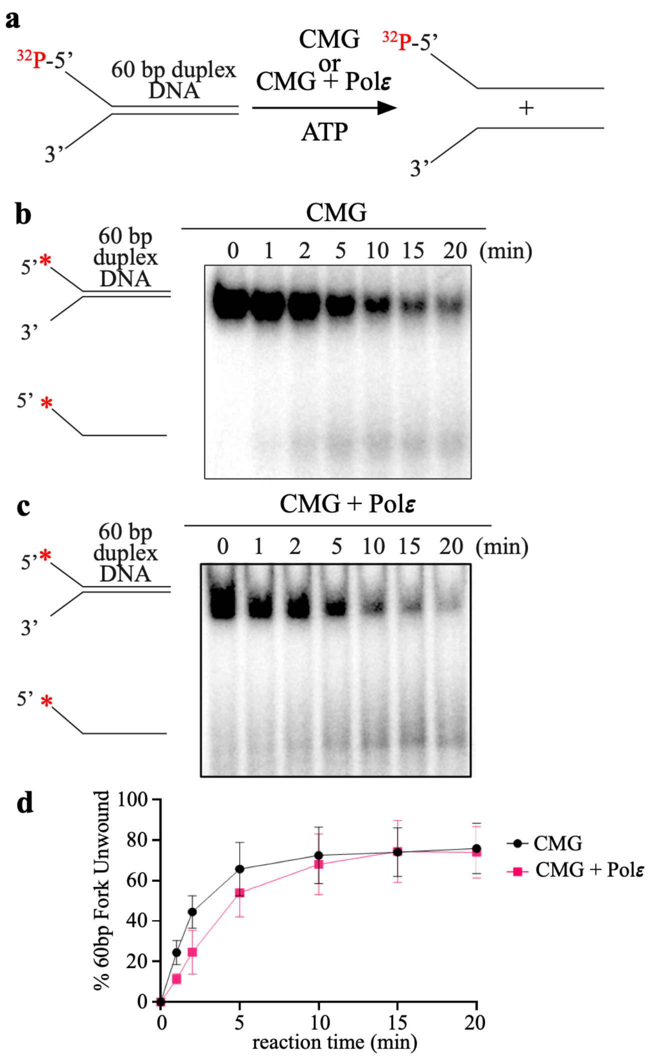

Supplementary Figure 10. The effect of Polε on CMG helicase activity *in vitro*.

(**a**) Schematic illustration of unwinding of a 60-bp forked DNA by CMG.

(**b-c**) Native PAGE of CMG helicase assays performed as in a in the absence (**b**) or presence (**c**) of Polε. (**d**) Quantification of the results. Data are presented as mean values +/- SEM (n=3). Source data are provided as a Source Data file.

Supplementary Table 1. Cryo-EM data collection, refinement, and validation statistics

Replisome					
	State I<br>EMD-37211<br>PDB: 8KG6	State II<br>EMD-37213<br>PDB: 8KG8	State III<br>EMD-37215<br>PDB: 8KG9	State IV<br>EMD-37345<br>PDB: 8W7S	State V<br>EMD-37343<br>PDB: 8W7M
Data Collection/Processing					
Voltage (kV)	300				
Magnification	81,000				
Defocus Range (µm)	-1 ~ -2.5				
Pixel Size (Å)	1.06				
Symmetry imposed	C1				
Total Electron Dose (e^-/Å^2)	53.13				
Exposure Time (s)	4.5				
Number of Images	21,776				
Number of Frames	40				
Initial Particle Number	2110,601				
Final Particle Number	384,519	147,795	17,199	7,939	28,871
Resolution (unmasked, Å)	4.1	7.8	8.7	13	7.7
Resolution (masked, Å)	3.07	4.23	4.52	7.29	4.12
FSC threshold	0.143				

Refinement	State I
**Model resolution (Å)**	3.3
**FSC threshold**	0.5
**Model composition**	
**Protein Residues**	8713
**Nucleotides**	57
**Ligand**	17
**B factor (Å^2)**	
**Protein**	140.66
**Nucleotide**	276.41
**Ligand**	76.39
**RMS Deviations**	
**Bond Lengths (Å)**	0.004
**Bond Angles (°)**	0.525
**Ramachandran**	
**Favored (%)**	95.00
**Allowed (%)**	5.00
**Outlier (%)**	0.00
**Molprobity Score**	1.85

Supplementary Table 2. Yeast strains used in this study

Yeast Strain	Genotype	Source
yJCZ2	*MATa / MATα ade2-1 ura3-1 his3-11,15 trp1-1 leu2-3,112 can1-100 bar1::Hyg pep4::KanMX his::HIS pRS303-CDC45iFLAG2 (pJY13) trp1::TRP1 pRS304-MCM4/MCM5 (pJF3) trp1::TRP1 pRS304-PSF1/SLD5 (pJCZ1) ura3:: URA3 pRS306-MCM2/3×FLAG-MCM3 (pJF6) leu2::LEU2 pRS305-MCM6/MCM7 (pJF4) leu2::LEU2pRS305-PSF2/PSF3 (pJCZ2)*	Zhou et al., 2017
yAE48	*MATa ade2-1 ura3-1 his3-11,15 trp1-1 leu2-3,112 can1-100 bar1::Hyg pep4::KanMX ura3::URA3 pRS306-CBP-TEV-CSM3/TOF1*	Yeeles et al., 2017
yAE31	*MATa ade2-1 ura3-1 his3-11,15 trp1-1 leu2-3,112 can1-100 bar1::Hyg pep4::KanMX his3::HIS3 pRS303-CBP-TEV-RFA1 ura3::URA3 pRS306-RFA2/RFA3*	Yeeles et al., 2015
yXZ1	*MATa ade2-1 ura3-1 his3-11,15 trp1-1 leu2-3,112 can1-100 bar1::Hyg pep4::KanMX trp:: TRP1 pRS404-DPB3/DPB4-TEV-HIS leu:: LEU2 pRS405-3×FLAG-TEV-DPB2 pESC-HIS-CBP-TEV-POL2*	This study
yXZ2	*MATa ade2-1 ura3-1 his3-11,15 trp1-1 leu2-3,112 can1-100 bar1::Hyg pep4::KanMX trp:: TRP1 pRS404-DPB3/DPB4-TEV-HIS leu:: LEU2 pRS405-3×FLAG-TEV-dpb2ΔNTD(Δ2-90) pESC-HIS-CBP-TEV-POL2*	This study
yXZ3	*MATa ade2-1 ura3-1 his3-11,15 trp1-1 leu2-3,112 can1-100 bar1::Hyg pep4::KanMX trp:: TRP1 pRS404-DPB3/ DPB4-TEV-HIS leu:: LEU2 pRS405-3×FLAG-TEV-dpb2ΔNTD(Δ2-90) pESC-HIS-CBP-TEV-pol2ΔDS2(Δ2023-2027Δ2069-2091)*	This study
yXZ4	*MATa ade2-1 ura3-1 his3-11,15 trp1-1 leu2-3,112 can1-100 bar1::Hyg pep4::KanMX trp:: TRP1 pRS404-DPB3/DPB4-TEV-HIS leu:: LEU2 pRS405-3×FLAG-TEV-dpb2ΔNTD(2-*	This study

	90) pESC-HIS-CBP-TEV-pol2ΔDS2+4(Δ1778-1784Δ2162-2186Δ2023-2027Δ2069-2091)	
yXZ5	*MATa ade2-1 ura3-1 his3-11,15 trp1-1 leu2-3,112 can1-100 bar1::Hyg pep4::KanMX trp:: TRP1 pRS404-DPB3/DPB4-TEV-HIS leu:: LEU2 pRS405-3×FLAG-TEV-DPB2 pESC-HIS-CBP-TEV-pol2ΔDS2(Δ2023-2027Δ2069-2091)*	This study
yXZ6	*MATa ade2-1 ura3-1 his3-11,15 trp1-1 leu2-3,112 can1-100 bar1::Hyg pep4::KanMX trp:: TRP1 pRS404-DPB3/DPB4-TEV-HIS leu:: LEU2 pRS405-3×FLAG-TEV-DPB2 pESC-HIS-CBP-TEV-pol2ΔDS2+4(Δ1778-1784Δ2162-2186Δ2023-2027Δ2069-2091)*	This study
YST2591	*MATa ade2-1 ura3-1 his3-11,15 trp1-1 leu2-3,112 can1-100 Δbar1::hisG SSN6::pST1760 (bidirectional promoter-tTA(TetR-VP16)_TetR'-SSN6, TDH3p-OsTIR1, HIS3) Δpep4::URA3 PSF2-3×FLAG-1×HA(LEU2) MCM7-13×myc (TRP1, KanMX)*	Miyazawa-Onami et al., 2017
YST3085	*MATa ade2-1 ura3-1 his3-11,15 trp1-1 leu2-3,112 can1-100 Δbar1::hisG leu2-3,112::pST1868 (bidirectional promoter between YBR188 & 189-tTA(TetR-VP16)_TetR'-SSN6, TDH3p-OsTIR1, LEU2) HphNT-TetO2-POL2-3×minidegron-KanMX MCM7-13×myc (TRP1, KanMX) PSF2-3×FLAG-1×HA(URA3) Δpep4::natNT1*	Miyazawa-Onami et al., 2017
yHYJ105	YST3085 *his3::HIS3 pRS403*	This study
yHYJ106	YST3085 his3::*HIS3 pRS403-POL2*	This study
yHYJ114	YST3085 *his::HIS3 pRS403 pol2ΔM2BD(Δ2069-2091)*	This study
yHYJ115	YST3085 *his::HIS3 pRS403 pol2ΔDS2(Δ2023-2027Δ2069-2091)*	This study
yHYJ109	YST3085 *his::HIS3 pRS403 pol2ΔM5BD(Δ1778-1784)*	This study
yHYJ109	YST3085 *his::HIS3 pRS403 pol2ΔZF2(Δ2162-2186)*	This study
yHYJ121	YST3085 *his::HIS3 pRS403 pol2ΔDS4(Δ1778-1784Δ2162-2186)*	This study

yHYJ120	YST3085 *his::HIS3 RS403 pol2ΔM5BD+M2BD(Δ1778-1784Δ2069-2091)*	This study
yHYJ122	YST3085 *his::HIS3 pRS403 Pol2ΔM2BD+ZF2(Δ2069-2091Δ2162-2186)*	This study
yHYJ117	YST3085 *his::HIS3 pRS403 pol2ΔDS2+4(Δ1778-1784Δ2023-2027Δ2069-2091Δ2162-2186)*	This study
