# Supplementary material for: Synergism between CMG helicase and leading strand DNA polymerase at replication fork
Source: Nat Commun. 2023 Sep 20;14:5849. doi: 10.1038/s41467-023-41506-0 (PMC10511561; doi:10.1038/s41467-023-41506-0)
Supplement: Supplementary file 3 — Description of Additional Supplementary Files [file 41467_2023_41506_MOESM3_ESM.pdf]

Description of Additional Supplementary Files

File Name: Supplementary Movie 1
Description: Mechanism coordinating DNA translocation by CMG and Polε docking to the motor domains of the MCM ring. Side (left) and MCM-CTD (right) views of the replisome illustrating its transition from State I to State V. The intermediate frames in between two states were interpolated by morphing. Subunits are color coded. Tof1-Csm3 and parental duplex DNA are not shown.
