# Supplementary material for: Synergism between CMG helicase and leading strand DNA polymerase at replication fork
Source: Nat Commun. 2023 Sep 20;14:5849. doi: 10.1038/s41467-023-41506-0 (PMC10511561; doi:10.1038/s41467-023-41506-0)
Supplement: Supplementary file 5 — Reporting Summary [file 41467_2023_41506_MOESM5_ESM.pdf]

nature research

Corresponding author(s): Shangyu Dang
Yuanliang Zhai

Last updated by author(s): Aug 31, 2023

Reporting Summary

Nature Research wishes to improve the reproducibility of the work that we publish. This form provides structure for consistency and transparency in reporting. For further information on Nature Research policies, see our Editorial Policies and the Editorial Policy Checklist.

Statistics

For all statistical analyses, confirm that the following items are present in the figure legend, table legend, main text, or Methods section.

n/a	Confirmed	
☐	☒	The exact sample size (*n*) for each experimental group/condition, given as a discrete number and unit of measurement
☒	☐	A statement on whether measurements were taken from distinct samples or whether the same sample was measured repeatedly
☒	☐	The statistical test(s) used AND whether they are one- or two-sided<br>*Only common tests should be described solely by name; describe more complex techniques in the Methods section.*
☒	☐	A description of all covariates tested
☒	☐	A description of any assumptions or corrections, such as tests of normality and adjustment for multiple comparisons
☐	☒	A full description of the statistical parameters including central tendency (e.g. means) or other basic estimates (e.g. regression coefficient) AND variation (e.g. standard deviation) or associated estimates of uncertainty (e.g. confidence intervals)
☒	☐	For null hypothesis testing, the test statistic (e.g. *F*, *t*, *r*) with confidence intervals, effect sizes, degrees of freedom and *P* value noted<br>*Give P values as exact values whenever suitable.*
☒	☐	For Bayesian analysis, information on the choice of priors and Markov chain Monte Carlo settings
☒	☐	For hierarchical and complex designs, identification of the appropriate level for tests and full reporting of outcomes
☒	☐	Estimates of effect sizes (e.g. Cohen's *d*, Pearson's *r*), indicating how they were calculated

Our web collection on statistics for biologists contains articles on many of the points above.

Software and code

Policy information about availability of computer code

Data collection	EPU software V2.10.0 was used for data collection with Titan Krios G3i (Thermo Fisher).
Data analysis	MotionCor2 v1.4.0, GCTF, Gautomatch, Relion 3.1, CryoSPARC v2.15.0, Pymol v1.8.X, UCSF Chimera v1.11.2 , Chimera X v1.3 COOT v0.9.5, Phenix v1.20

For manuscripts utilizing custom algorithms or software that are central to the research but not yet described in published literature, software must be made available to editors and reviewers. We strongly encourage code deposition in a community repository (e.g. GitHub). See the Nature Research guidelines for submitting code & software for further information.

Data

Policy information about availability of data

All manuscripts must include a data availability statement. This statement should provide the following information, where applicable:

- Accession codes, unique identifiers, or web links for publicly available datasets
- A list of figures that have associated raw data
- A description of any restrictions on data availability

Cryo-EM density maps of the replisome complexes have been deposited in the Electron Microscopy Data Bank (accession no. EMD-37211, MD-37213 , EMD-37215, EMD-37345, and EMD-37343). Atomic coordinate has been deposited in the Protein Data Bank (ID codes: 8KG6, 8KG8, 8KG9, 8W7S and 8W7W). All functional data generated or analyzed during this study are included in the published paper.

Field-specific reporting

Please select the one below that is the best fit for your research. If you are not sure, read the appropriate sections before making your selection.

☒ Life sciences ☐ Behavioural & social sciences ☐ Ecological, evolutionary & environmental sciences

For a reference copy of the document with all sections, see nature.com/documents/nr-reporting-summary-flat.pdf

Life sciences study design

All studies must disclose on these points even when the disclosure is negative.

Sample size	No statistical methods were used to predetermine sample size. All biochemical experiments were replicated two or more times. For structural data, the number of micrographs collected was determined based on the target resolution of the relevant electron microscopy 3D structures.
Data exclusions	Regarding the cryo-EM raw micrograph screening, exclusion was done based on the quality of the images. Regarding the particle selection, 2D and 3D classification were used and criterion is based on the quality of resulting 2D class average and 3D maps.
Replication	All attempts of replication were successful. Cryo-EM single particle analysis relies on averaging over a large number of independent observations.
Randomization	Samples were not allocated to groups.
Blinding	Investigators were not blinded during data acquisition and analysis because because visual inspection is necessary for the methods employed.

Reporting for specific materials, systems and methods

We require information from authors about some types of materials, experimental systems and methods used in many studies. Here, indicate whether each material, system or method listed is relevant to your study. If you are not sure if a list item applies to your research, read the appropriate section before selecting a response.

Materials & experimental systems

n/a	Involved in the study	
☐	☒	Antibodies
☐	☒	Eukaryotic cell lines
☒	☐	Palaeontology and archaeology
☒	☐	Animals and other organisms
☒	☐	Human research participants
☒	☐	Clinical data
☒	☐	Dual use research of concern

Methods

n/a	Involved in the study	
☒	☐	ChIP-seq
☐	☒	Flow cytometry
☒	☐	MRI-based neuroimaging

Antibodies

Antibodies used	anti-Flag (1:1000, Cell Signaling Technology, #14793), anti-Cdc45 (1:5000, a gift from Bruce Stillman), and anti-Mcm2 (1:1000, a gift from Bruce Stillman).
Validation	Anti-Flag antibody (#14793), commercially available in Cell Signaling Technology. It can be used for DYKDDDDK Tag (D6W5B) Rabbit mAb detects exogenously expressed DYKDDDDK proteins in cells. The antibody recognizes the DYKDDDDK peptide, which is the same epitope recognized by Sigma's Anti-FLAG® antibodies, fused to either the amino-terminus or carboxy-terminus of the target protein. This antibody has been validated by the vendor.<br>Anti-Cdc45 and anti-Mcm2 antibodies are gifts from Bruce Stillman (Cold Spring Harbor Laboratory). Western blots were also done to validate the specificity of antibodies towards Mcm2 and Cdc45.

Eukaryotic cell lines

Policy information about cell lines

Cell line source(s)	Yeast W303-1a
Authentication	The cells were used only for protein purification and functional assays, and not further authenticated.
Mycoplasma contamination	The cell line for purification was not tested.

Commonly misidentified lines (See ICLAC register)	No cell line used in this study was commonly misidentified lines.

Flow Cytometry

Plots

Confirm that:

- [ ] The axis labels state the marker and fluorochrome used (e.g. CD4-FITC).
- [x] The axis scales are clearly visible. Include numbers along axes only for bottom left plot of group (a 'group' is an analysis of identical markers).
- [ ] All plots are contour plots with outliers or pseudocolor plots.
- [ ] A numerical value for number of cells or percentage (with statistics) is provided.

Methodology

Sample preparation	Ethanol fixed yeast cells were washed with and resuspended in sodium citrate solution. RNase and Proteinase K were added sequentially to remove RNAs and proteins. DNA was stained with PBS buffer (pH 7.4; 137 mM NaCl, 2.7 mM KCl, 10 mM Na2HPO4, 1.8 mM KH2PO4) containing 0.5 mg/ml Propidium iodide (Sigma, P4170).
Instrument	BD FACSAriaIII Flow Cytometer
Software	FlowJo software
Cell population abundance	NA
Gating strategy	NA

- [ ] Tick this box to confirm that a figure exemplifying the gating strategy is provided in the Supplementary Information.
